# Clinical significance of glypican-3-positive circulating tumor cells of hepatocellular carcinoma patients: A prospective study

**DOI:** 10.1371/journal.pone.0217586

**Published:** 2019-05-29

**Authors:** Michinori Hamaoka, Tsuyoshi Kobayashi, Yuka Tanaka, Hiroaki Mashima, Hideki Ohdan

**Affiliations:** Department of Gastroenterological and Transplant Surgery, Graduate School of Biomedical and Health Sciences, Hiroshima University, Hiroshima, Japan; University of Navarra School of Medicine and Center for Applied Medical Research (CIMA), SPAIN

## Abstract

The utility of glypican-3 (GPC3) expression for the detection of circulating tumor cells (CTCs) in hepatocellular carcinoma (HCC) patients has not been elucidated. The aim of this study was to identify associations between the presence of GPC3-positive CTCs and clinicopathological factors of these patients, furthermore, to evaluate whether CTC can predict microscopic portal vein invasion (mPVI). This study was done on 85 patients who underwent hepatectomy as the first-line treatment and whose preoperative imaging showed no evidence of macroscopic PVI and distant metastases. Peripheral blood was collected from all patients immediately before surgery. Cells were purified initially by density gradient centrifugation followed by immunomagnetic positive enrichment based upon the expression of GPC3. The numbers of CTCs contained in the enriched samples were enumerated via flow cytometry. Protocol validation using HepG2 cells spiked into 8.0 mL of blood from a healthy volunteer indicated that we were able to recover 12.1% of the tumor cells. A median number of 3 CTCs (range: 0–27) was detected in the 8.0 mL of peripheral blood of the 85 analyzed HCC patients. Thirty-three patients had CTCs ≥5, and these patients had a higher incidence of mPVI (p < 0.001), a lower disease-free survival (p = 0.015), and a lower overall survival (p = 0.047) than those with CTCs <5. Multivariate analysis identified CTCs ≥5 as an independent predictor of mPVI (p < 0.001). In conclusion, preoperative GPC3-positive CTCs ≥5 was a risk factor of mPVI and poor prognosis, and therefore may be a useful biomarker for HCC patient outcomes.

## Introduction

Although hepatectomy is expected to be curative for hepatocellular carcinoma (HCC), tumor recurrence, most of which occurs in the remnant liver, is common in these patients. Indeed, approximately 30% of these patients develop recurrence within the first year after surgery, and early recurrence is one of the most important prognostic factors for this disease [1]. Tumor invasion in the portal vein, even if it is microscopic portal vein invasion (mPVI), is associated with increased risk leading to early recurrences. Although anatomical liver resection should be considered for the patients with mPVI, it is difficult to detect mPVI before the treatment of HCC [2–5]. Therefore, more reliable marker of mPVI is needed.

Circulating tumor cells (CTCs) are cancer cells that shed from either a primary or metastatic tumor, which then circulate in the peripheral blood. Although the presence of CTCs in HCC has been reported in several studies, several challenges that must be overcome remain. For both HCC and other types of cancer, the current standard method for the detection of CTCs in the clinical setting is positive immuno-magnetic bead separation using antibodies that recognize tumor cell surface antigens. Epithelial cell adhesion molecule (EpCAM) is an epithelial cell-specific surface marker widely expressed on both normal and tumor epithelium [6]. Although HCC cells are indeed epithelial cells, only approximately 35% of HCC cases express EpCAM [7, 8], and EpCAM-positive CTCs are detected only in approximately 20%–40% of patients [9–12]. An alternative method is a negative depletion CTCs enrichment that relies on the removal of normal cells using immunomagnetic separation in the blood followed by immunofluorescence or image flow cytometer to detect CTCs expressing epithelial markers such as EpCAM and cytokeratin [13–15]. Therefore, identification of tumor cell surface antigens that facilitate CTC detection in HCC patients represents an unmet medical need. Although numerous studies have demonstrated that the presence of CTCs was significantly associated with prognosis and other clinicopathologic features of HCC [9–14, 16, 17], the utility of CTCs for predicting mPVI has not been studied.

Glypican-3 (GPC3) is an oncofetal heparan sulfate proteoglycan that is currently used as a diagnostic biomarker for distinguishing HCC from normal liver tissue, benign hepatic tumors, and other types of metastatic carcinomas [18, 19]. GPC3 is expressed in 70–90% of primary HCCs, wherein it is localized to the cytoplasm and/or cell membrane [20], is observed less frequently in well-differentiated HCCs than in moderately and poorly differentiated HCCs [18, 20], and is an indicator of disease prognosis [20–22]. However, the utility of GPC3 expression for the detection of CTCs in HCC patients has not been elucidated.

The aim of this study was to develop a method by which to use GPC3 expression for the detection of living CTCs. In addition, we sought to identify associations between GPC3-positive CTCs and clinicopathological factors of HCC patients. Finally, we evaluated the utility of GPC3-positive CTCs for predicting mPVI.

## Materials and methods

### Study design

HCC patients (n = 85) who underwent hepatectomy as the first-line treatment between April 2015 and October 2016 at Hiroshima University Hospital, Hiroshima, Japan were enrolled in this study. None of the patients had distant metastases and had not yet begun treatment for cancer at the time of enrollment. Based on the predictors of mPVI in clinical setting, the patients were excluded due to the presence of macroscopic PVI on preoperative imaging. Clinicopathological findings were recorded according to the criteria established by the Liver Cancer Study Group of Japan. Microscopic PVI was defined as microscopically observable tumor invasion of (or tumor thrombus in) distal to the second-order branches of the portal vein but not of the second-order branches. This prospective, single-institution study was conducted in accordance with the 1975 Declaration of Helsinki. The study was approved by the institutional review board of Hiroshima University, Hiroshima, Japan (approval number: E-320), and was registered with the University Hospital Medical Information Network Clinical Trial Registry (UMIN-CTR), Japan (registration number: UMIN000025989). Written informed consent was obtained from each patient.

### Identification and analysis of CTCs

Peripheral blood was collected from 85 patients with HCC immediately before surgery, as well as from 27 individuals without HCC (12 healthy volunteers, 4 cirrhotic patients, 4 patients with benign liver tumors, 5 patients with malignant liver tumors other than HCC, and 2 patients with colorectal cancer). CTCs were detected by immuno-magnetic positive enrichment coupled with flow cytometry. Briefly, the mononuclear cell layer of each blood sample, which included CTCs, was isolated by density gradient centrifugation, followed by immuno-magnetic positive selection to recover GPC3-positive cells. The numbers of CTCs contained in the enriched samples were enumerated via flow cytometry by independent technicians who were blinded to all clinicopathological factors and patient outcomes.

### Mononuclear cell isolation

Mononuclear cells, including CTCs, were isolated from whole blood samples by density gradient centrifugation according to the manufacturer’s instructions. Briefly, 8.0 mL of blood was drawn from a peripheral vessel, and collected into vacuum blood collection tubes containing sodium citrate, a polyester gel, and a density gradient liquid (BD Vacutainer CPT Cell Preparation Tube). The samples were centrifuged at 1800 × g for 20 min at 25°C. Samples were processed within 2 hours of their collection.

### Immunomagnetic enrichment

The mononuclear cell pellet of each sample was resuspended in MACS buffer (autoMACS Running Buffer, Miltenyi Biotec, Germany). Samples were then incubated with allophycocyanin (APC)-conjugated mouse anti-human GPC3 monoclonal antibodies (clone #307801; R&D Systems Inc., USA) for 30 min at 4°C in the dark. Cells were then washed with MACS buffer and centrifuged at 300 × g for 10 min at 4°C. The supernatant was aspirated and the remaining cells were resuspended in MACS buffer. Samples were then incubated with anti-APC MicroBeads (Miltenyi Biotec) for 15 min at 4°C in the dark. Cells were again washed with MACS buffer and centrifuged at 300 × g for 5 min at 4°C. The supernatant was aspirated and the remaining cells were resuspended in MACS buffer. GPC3-positive cells were sorted using an automated magnetic cell sorter (autoMACS Separator, Miltenyi Biotec). Cells in the positive fraction were collected and used in further analyses.

### Immunofluorescent staining

To identify CTCs on slides, immunofluorescence experiment was based on that described in a previous report [23]. Briefly, GPC3-positive cell fractions were centrifuged at 1500 rpm for 3 min onto poly-lysine-coated slides. The slides were fixed for 30 min in 4% paraformaldehyde and rinsed with washing buffer (phosphate buffered saline (PBS) with 0.05% TWEEN 20). Non-specific binding sites were then blocked with 5% bovine serum albumin (BSA) in PBS for 30 min, and slides were incubated with mouse monoclonal anti-cytokeratin7/8 antibodies (clone CAM5.2; prediluted; Becton Dickinson, Franklin Lakes, NJ, USA) to identify cancer cells and rabbit monoclonal anti-human CD45 antibodies (clone D9M8I; dilution 1/200; Cell Signaling Technology, Danvers, MA, USA) to identify white blood cells for 1 hour at room temperature in a humidified chamber. A BD Horizon BV480-conjugated goat anti-mouse IgG (Becton Dickinson) and an Alexa Fluor 555-conjugated goat anti-rabbit IgG (heavy + light chains) (Thermo Fisher Scientific, Waltham, MA, USA) served as secondary antibodies. Slides were mounted with Vectashield mounting medium with 4’,6-diamino-2-phenylindole (DAPI) (Vector Laboratories, Burlingame, CA, USA) to stain cell nuclei. Immunofluorescence images were capture using a BZ-9000 fluorescent microscope (Keyence, Japan).

### Flow cytometric analysis

Immunomagnetically enriched samples were analyzed via flow cytometry. Briefly, samples were incubated with phycoerythrin-cyanine 7 (PE-Cy7)-conjugated mouse anti-human CD45 antibodies (clone HI30; Becton Dickinson) and fluorescein isothiocyanate (FITC)-conjugated mouse anti-human CD235a antibodies (clone GA-R2 (HIR2); Becton Dickinson) to identify erythroid cells for 30 min at 4°C in the dark. Cells were washed with flow cytometry staining buffer (PBS with 0.1% BSA and 0.1% sodium azide) and centrifuged at 2000 rpm for 5 min. The supernatant was aspirated, and the remaining cells were resuspended in FACS buffer. For the detection of dead cells, 7-Aminoactinomycin D (7-AAD, Becton Dickinson) was added to the buffer immediately prior to flow cytometry. In this study, we considered the GPC3+CD45-CD235a-7-AAD- events detected by flow cytometry as CTCs. Flow cytometry was carried out on BD FACS Canto II (Becton Dickinson, USA), and the data were analyzed with FlowJo version 7.6.5 (Tree Star Inc., Ashland, OR, USA).

### Detection of spiked tumor cells

GPC3-positive HepG2 human HCC cells obtained from the American Type Culture Collection (ATCC) were used in a spiking experiment to evaluate the recovery and linearity of our GPC3+CD45-CD235a-7-AAD- CTC detection method. HepG2 cells were harvested by incubation with 0.05% trypsin for 1 min at 37°C. Cells were washed with and resuspended in PBS at a density of 1 × 10^4^ cells per mL; cell density was confirmed with flow cytometry. This suspension was serially diluted to the required cell densities. A defined number of HepG2 cells (range: 10–500 cells) was spiked into 8.0 mL of peripheral blood from a healthy volunteer, and samples were processed as described above. Briefly, GPC3-positive cells, or HepG2 cells, were enumerated via flow cytometry after density gradient centrifugation and immuno-magnetic positive enrichment based upon expression of GPC3.

### Statistics

All statistical analyses were performed using JMP 11 software (SAS Institute Inc., Cary, NC, USA). The Wilcoxon rank-sum test was used to compare continuous variables, and categorical variables were compared using the chi-squared or Fisher’s exact probability test, as appropriate. Disease-free survival rates and overall survival rates were calculated using the Kaplan-Meier method and were compared using the log-rank test. The receiver operating characteristic (ROC) curve was used to determine the cutoff value for each continuous variable. In multivariate analysis, a multiple logistic regression model was employed to identify independent predictors of mPVI. Only variables that were statistically significant in univariate analyses were included in the multivariate analysis. Spearman’s correlation coefficient was used to estimate the linear relationship between the numbers of spiked and detected HepG2 cells in each sample. For all analyses, p-values < 0.05 were considered significant. All relevant data are shown within Supporting Information file (S1 Table).

## Results

### CTC detection method

**Fig 1:**
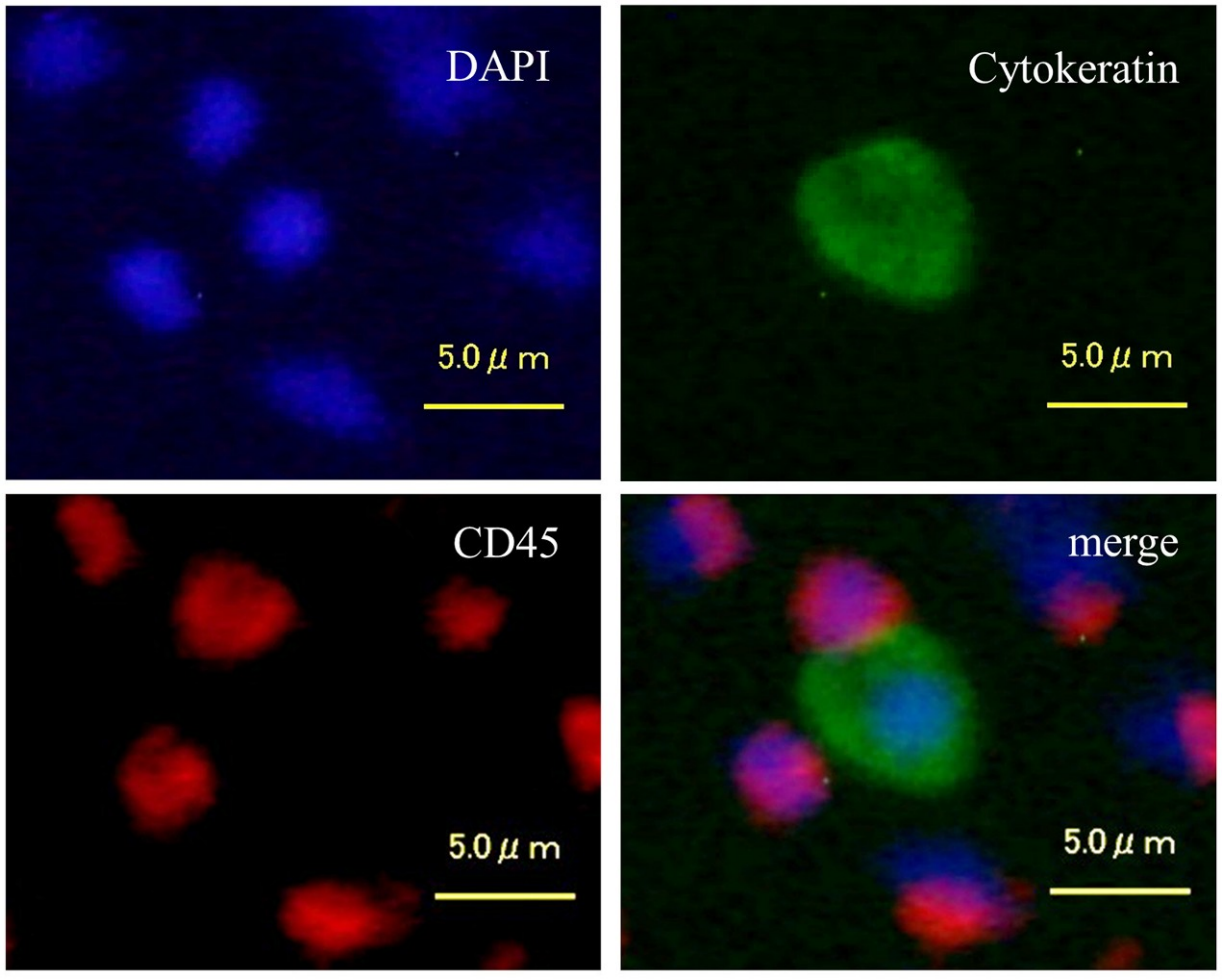
Fluorescence microscopy images of cells isolated from peripheral blood of HCC patients by positive immunomagnetic separation. Images of cells stained with DAPI (blue), anti-cytokeratin antibodies (green), and CD45 antibodies (red) and the corresponding merged image are shown. Cytokeratin-positive cells are CTCs, whereas CD45-positive cells are lymphocytes.

**Fig 2:**
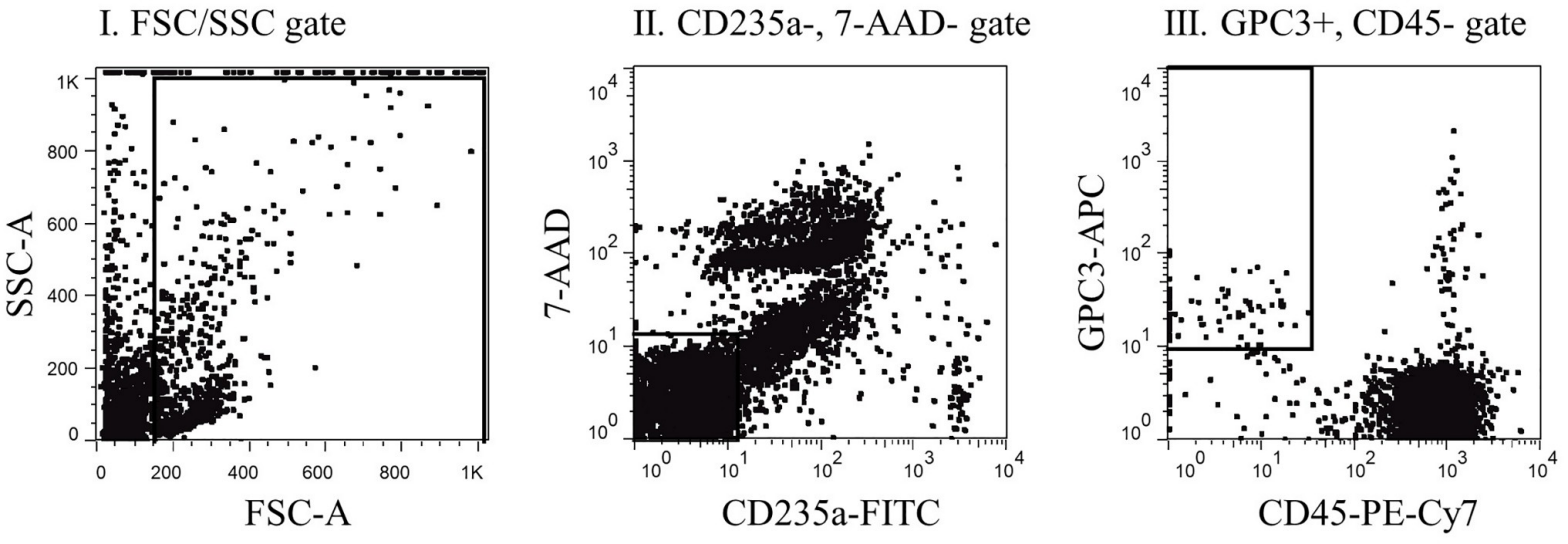
Gating strategy. The first step represents the forward-scatter (FSC) versus side-scatter (SSC) plot (left). In this step, the unassigned events and artifacts (including cell debris, mechanical noise, and cell clumps) are gated off. In the second step, the dead cells and CD235a-positive cells are gated off on the CD235a versus 7-AAD plot (middle). In the remaining CD45-negative cells, GPC3-positive cells are selected with the CD45 versus GPC3 plot (right).

GPC3-positive cells were isolated from the mononuclear cell layer of each blood sample using positive immuno-magnetic separation. Subsequently, GPC3-positive mononuclear cells were centrifuged and placed onto a slide glass and subjected to fluorescent immunostaining to confirm the presence of CTCs. A cell was classified as a CTC when it exhibited a round or oval morphology with a visible nucleus, and displayed epithelial characteristics, such as cytokeratin7/8 and DAPI positivity, as well as CD45 negativity (Fig 1). Using fluorescent immunostaining, we confirmed that CTCs were contained within the immuno-magnetically enriched samples. However, we also found that a considerable number of CD45-positive cells was also present within these samples. Therefore, the enriched cell suspension was analyzed via flow cytometry to exclude CD45-positive and CD235a-positive blood cells, as well as dead cells incorporating 7-AAD. The gating strategy for the detection of GPC3^+^CD45^-^CD235a^-^7-AAD^-^ CTCs is presented in Fig 2.

### Validation of CTC detection method: Recovery and linearity

**Fig 3:**
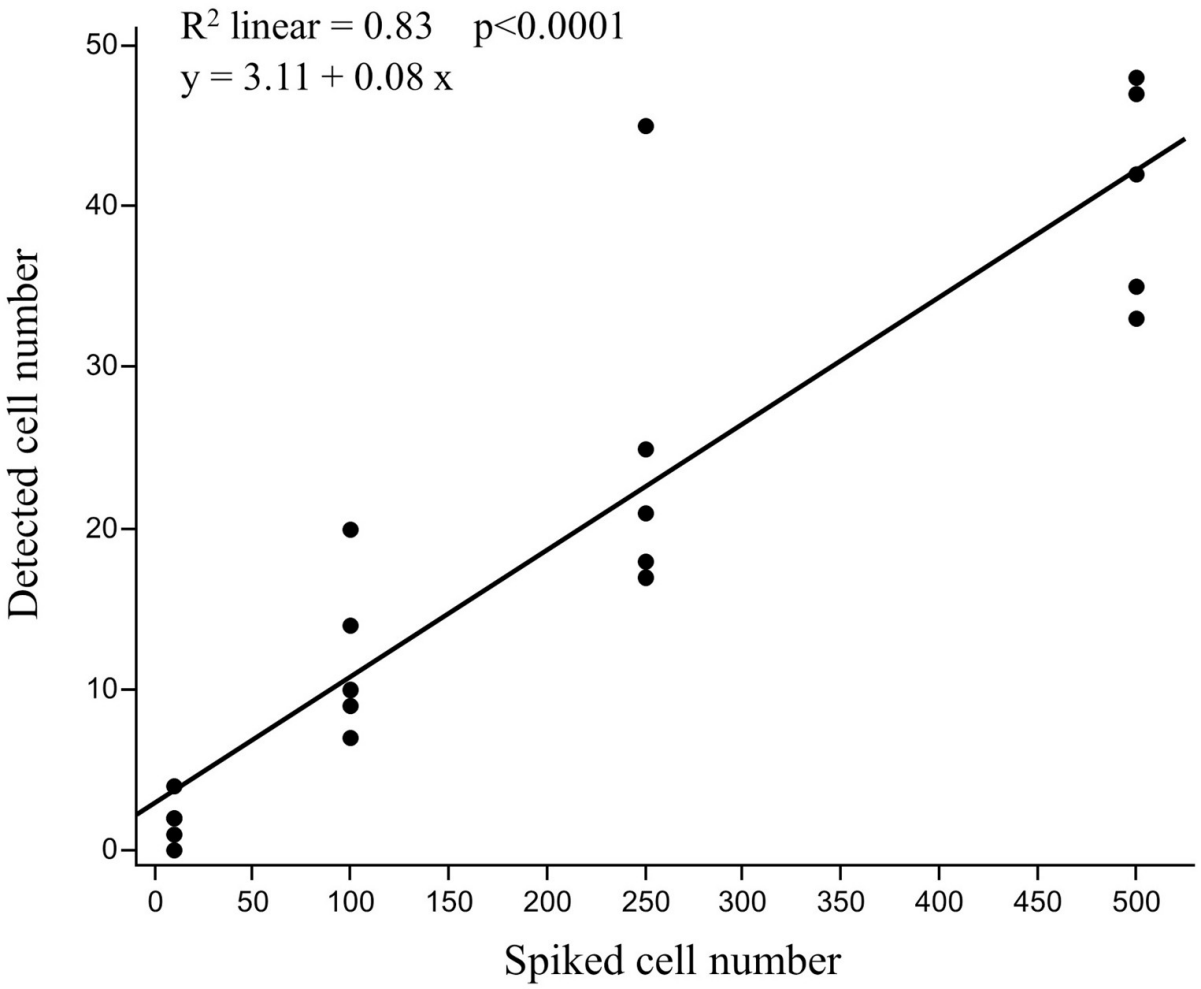
Linear correlation between the numbers of spiked and detected HepG2 cells. HepG2 cells (10, 100, 250, and 500 cells) were spiked into 8.0 mL of blood from a healthy volunteer. The number of cells spiked (x-axis) is plotted versus the number of cells detected by flow cytometry (y-axis), with the correlation coefficient shown at the top of the graph.

To validate our CTC detection method, GPC3-positive HepG2 cells were spiked into 8.0 mL of healthy volunteer blood and isolated using our GPC3-positive cell recovery protocol. The number of spiked HepG2 cells was plotted against the number of HepG2 cells detected by flow cytometry in each sample (Fig 3). Linear regression analysis yielded a slope of 0.08 (95% confidence interval [CI]: 0.06–0.10), an intercept of 3.11 (95% CI: -1.97–8.18), and a correlation coefficient (R^2^) of 0.83. In addition, the average spiked cell recovery rate, calculated as the number of spiked cells recovered/total number of spiked cells, was 12.1% ± 4.2% across all spiked cell numbers tested (range: 10–500). Therefore, since the numbers of spiked and detected tumor cells were positively correlated, we concluded that our method accurately recognized tumor cells.

### Associations between patient characteristics and presence of CTCs

**Fig 4:**
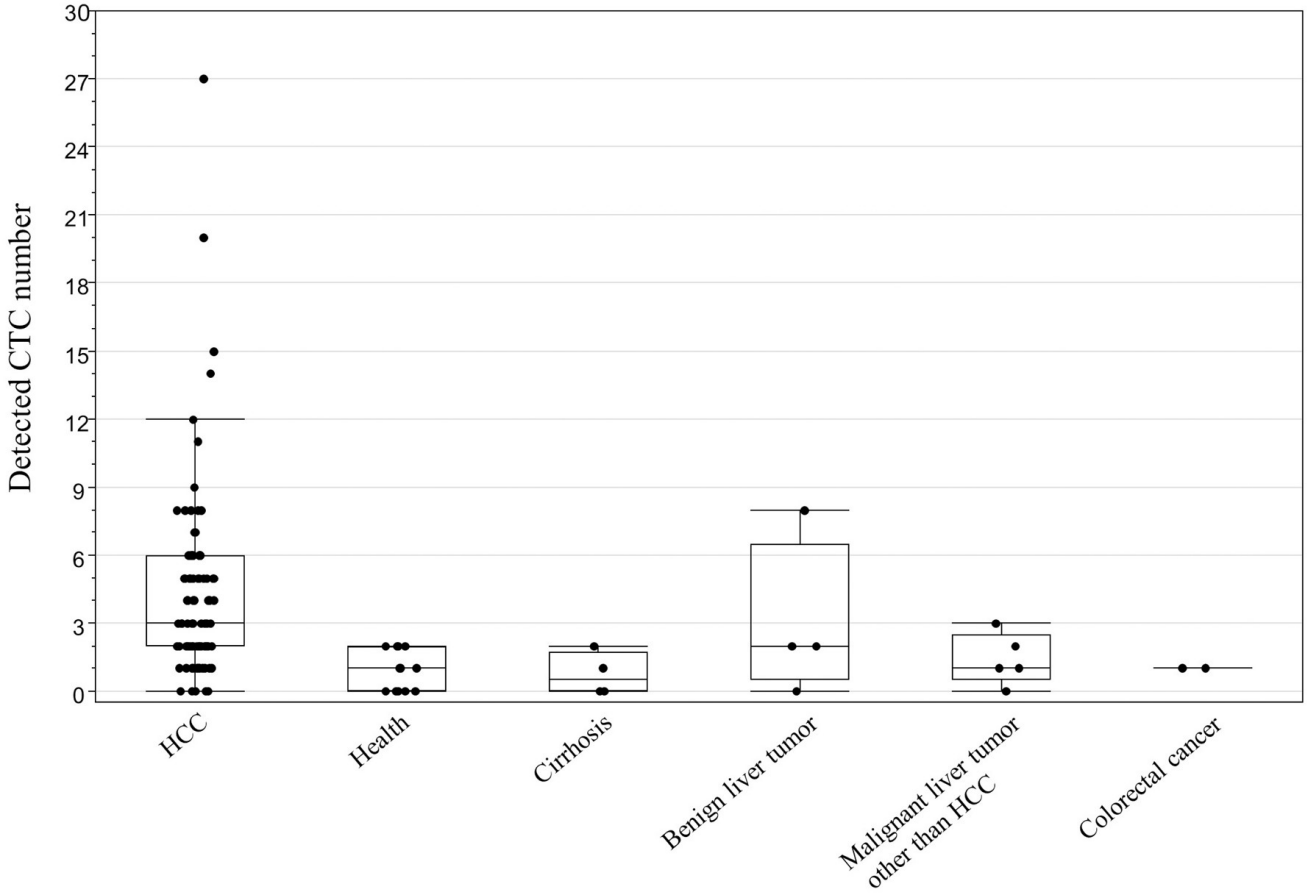
Dot and box-plots of GPC3-positive CTC count. Distribution of GPC3-positive CTCs in HCC patients (n = 85), healthy volunteers (n = 12), cirrhotic patients (n = 4), patients with benign liver tumors (n = 4), patients with malignant liver tumors other than HCC (n = 5), and patients with colorectal cancer (n = 2).

**Fig 5:**
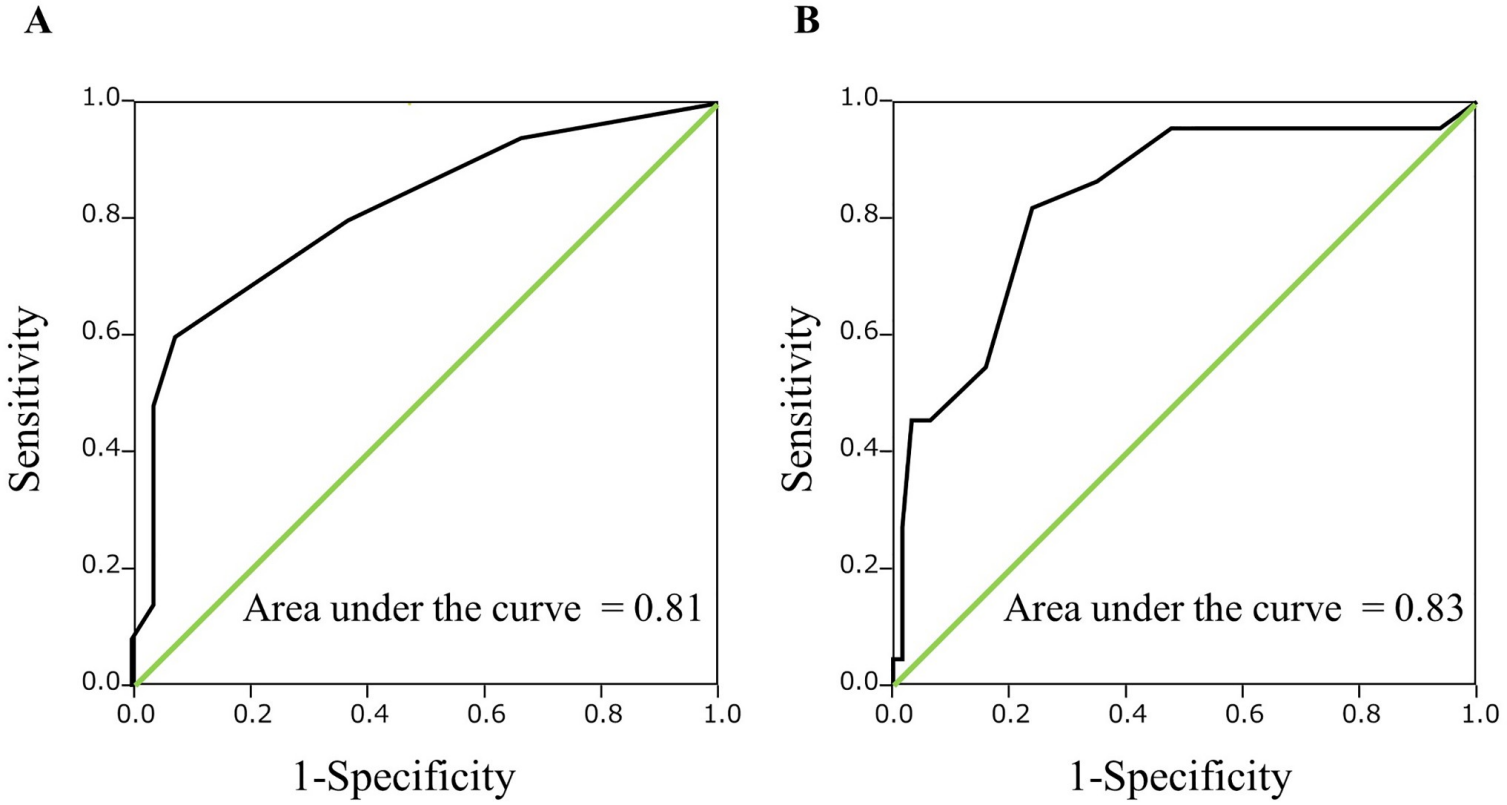
(A) ROC curve of CTC count for discrimination between HCC patients and non-HCC patients. (B) ROC curve of CTC count for prediction of microscopic portal vein invasion in HCC patients.

Patient demographic and clinicopathological information is summarized in Table 1. A median number of 3 CTCs (range: 0–27) was detected by flow cytometry in the peripheral blood of the 85 analyzed HCC patients. In addition, a median number of 1 CTC (range: 0–8) was detected in the peripheral blood of the 27 individuals without HCC. The distribution of data in different comparator groups was shown in Fig 4. The area under the ROC curve for CTC count in discrimination between HCC patients and non-HCC patients was 0.81. ROC curve analysis inclusive of all subjects indicated that a value for the number of CTCs ≥3 could be considered a promising tumor marker for HCC, with a sensitivity and specificity of 60.0% and 92.6%, respectively (Fig 5A). In addition, ROC curve analysis in 85 HCC patients identified a cutoff of 5 could be considered a promising surrogate biomarker of mPVI, with a sensitivity and specificity of 81.8% and 76.2%, respectively (Fig 5B). From this result, HCC patients were then divided into two groups: a CTC ≥5 group (n = 33), and a CTC <5 group (n = 52). Table 2 compares the clinicopathological features of these two groups. Patients in the CTC ≥5 group had significantly larger tumor size (p = 0.012), a greater number of tumors (p = 0.026), a greater number of encapsulated HCC cases (p = 0.004), a higher incidence of mPVI (p < 0.001), a higher incidence of hepatic vein invasion (p = 0.020), and a greater number of poorly differentiated HCC cases (p = 0.013) than patients in the CTC <5 group.

**Table 1:** Clinicopathologic features.

Variables	HCC patients (*n* = 85)
Age (years)	71 (47–87)
Sex; male / female: *n* (%)	68 (80) / 17 (20)
BMI (kg/m^2^)	23.6 (17.0–33.3)
HBV / HCV / NBNC: *n* (%)	13 (15) / 37 (44) / 35 (41)
Prothrombin activity (%)	82 (24–106)
Total bilirubin (mg/dl)	0.8 (0.3–3.7)
AST (IU/L)	33 (12–141)
ALT (IU/L)	27 (9–242)
Albumin (g/dl)	3.9 (2.6–5.2)
ICGR15 (%)	12.9 (2.5–54.6)
Child-Pugh grade; A / B: *n* (%)	75 (88) / 10 (12)
AFP (ng/ml)	7.6 (1.1–290700)
AFP-L3 (%)	5 (0–83)
DCP (mAU/ml)	68 (10–838500)
Tumor number	1 (1–10)
Tumor size (mm)	25 (4–230)
Tumor encapsulation: *n* (%)	66 (78)
Microscopic portal vein invasion: *n* (%)	22 (26)
Hepatic vein invasion: *n* (%)	6 (7)
Differentiation; well / mod. / por.: *n* (%)	14 (16) / 60 (71) / 11 (13)
The number of CTCs (cells)	3 (0–27)

Data are expressed as median (range) for continuous and n (%) for categorical variables. BMI:body mass index, HBV:Hepatitis B surface antigen positive, HCV:Hepatitis C antibody positive, NBNC:non-HBV and non-HCV, AST: aspartate aminotransferase, ALT: alanine aminotransferase, ICGR15: indocyanine green 15-min retention test, AFP:alpha-fetoprotein, AFP-L3:lectin-reactive alpha-fetoprotein, DCP:des-gamma-carboxy prothrombin, mod.: moderate, por.: poorly, CTCs: circulating tumor cells.

**Table 2:** Clinicopathologic features between CTC ≥5 and <5 group.

Variables	CTC ≥5 group	CTC <5 group	*P*-value
	(*n* = 33)	(*n* = 52)	
Age (years)	69 (48–87)	71 (47–82)	0.244
Male: *n* (%)	27 (82)	41 (79)	0.739
BMI (kg/m^2^)	24.5 (18.6–33.2)	23.4 (17.0–33.3)	0.248
HBV: *n* (%)	4 (12)	9 (17)	0.517
HCV: *n* (%)	13 (39)	24 (46)	0.540
NBNC: *n* (%)	16 (48)	19 (37)	0.275
Prothrombin time (%)	78 (61–101)	84 (24–106)	0.016
Total bilirubin (mg/dl)	0.8 (0.3–2.1)	0.9 (0.4–3.7)	0.986
AST (IU/L)	33 (12–106)	32 (12–141)	0.579
ALT (IU/L)	24 (11–52)	30 (9–242)	0.227
Albumin (g/dl)	3.8 (2.6–5.2)	4.0 (2.7–4.9)	0.016
ICGR15 (%)	15.4 (2.6–54.6)	12.1 (2.5–38.8)	0.570
Child-Pugh grade B: *n* (%)	6 (18)	4 (8)	0.144
AFP (ng/ml)	10.4 (1.8–12772)	6.4 (1.1–290700)	0.184
AFP-L3 (%)	8.0 (0.5–78)	3.5 (0–83)	0.026
DCP (mAU/ml)	364 (10–838500)	47 (10–49989)	0.160
Tumor number	1 (1–10)	1 (1–5)	0.026
Tumor size (mm)	33 (7–230)	25 (4–180)	0.012
Tumor encapsulation: *n* (%)	31 (94)	35 (67)	0.004
Microscopic portal vein invasion: n (%)	18 (55)	4 (8)	<0.001
Hepatic vein invasion: *n* (%)	5 (16)	1 (2)	0.020
Well differentiated: *n* (%)	4 (12)	10 (19)	0.389
Moderately differentiated: *n* (%)	21 (64)	39 (75)	0.263
Poorly differentiated: *n* (%)	8 (24)	3 (6)	0.013

Data are expressed as median (range) for continuous and n (%) for categorical variables. CTC: circulating tumor cell, BMI:body mass index, HBV:Hepatitis B surface antigen positive, HCV:Hepatitis C antibody positive, NBNC:non-HBV and non-HCV, AST: aspartate aminotransferase, ALT: alanine aminotransferase, ICGR15: indocyanine green 15-min retention test, AFP:alpha-fetoprotein, AFP-L3:lectin-reactive alpha-fetoprotein, DCP:des-gamma-carboxy prothrombin.

### Correlation between peripheral blood CTCs and incidence of microscopic portal vein invasion

Univariate analysis showed that CTCs ≥5 (p < 0.001), prothrombin activity <80% (p = 0.038), albumin level <3.5 g/dL (p = 0.002), alpha-fetoprotein (AFP) level ≥60 ng/mL (p = 0.001), lectin-reactive alpha-fetoprotein (AFP-L3) value ≥10% (p = 0.001), and multiple tumors (p = 0.048) were associated with mPVI. Subsequent multivariate analysis identified CTCs ≥5 (odds ratio [OR]: 14.60; 95% CI: 3.27–106.14; p < 0.001) and AFP level ≥60 ng/mL (OR: 9.35; 95% CI: 1.68–76.88; p = 0.010) as independent risk factors for mPVI (Table 3).

**Table 3:** Preoperative factors associated with microscopic portal vein invasion.

Variables	Univariate analysis		Multivariate analysis	
	OR (95% CI)	*P*-value	OR (95% CI)	*P*-value
Age < 75 years	1.43 (0.49–4.18)	0.511		
Male	0.80 (0.25–2.60)	0.710		
BMI ≥ 24.5 kg/m^2^	1.74 (0.65–4.64)	0.266		
Prothrombin activity < 80%	2.84 (1.04–7.78)	0.038	1.62 (0.37–7.15)	0.509
Total bilirubin ≥ 1.0 mg/dl	1.14 (0.41–3.15)	0.796		
AST ≥ 30 IU/L	0.66 (0.25–1.75)	0.399		
ALT ≥ 30 IU/L	0.81 (0.30–2.17)	0.677		
Albumin < 3.5 g/dl	5.54 (1.74–17.62)	0.002	1.42 (0.18–9.95)	0.730
ICGR15 ≥ 10%	1.53 (0.53–4.47)	0.432		
Child-Pugh grade B	3.41 (0.88–13.19)	0.064		
AFP ≥ 60 ng/ml	6.55 (2.07–20.68)	0.001	9.35 (1.68–76.88)	0.010
AFP-L3 ≥ 10%	6.14 (2.03–18.61)	0.001	2.00 (0.47–8.64)	0.347
DCP ≥ 100 mAU/ml	1.82 (0.69–4.86)	0.226		
Multiple tumors	2.71 (0.99–7.39)	0.048	1.13 (0.23–5.33)	0.878
Tumor size ≥ 30 mm	2.35 (0.87–6.32)	0.226		
CTC ≥5	14.4 (4.21–49.21)	<0.001	14.60 (3.27–106.14)	<0.001

OR:odds ratio, CI:confidence interval, BMI:body mass index, AST: aspartate aminotransferase, ALT: alanine aminotransferase, ICGR15: indocyanine green 15-min retention test, AFP:alpha-fetoprotein, AFP-L3:lectin-reactive alpha-fetoprotein, DCP:des-gamma-carboxy prothrombin, CTC: circulating tumor cell.

### Associations between CTCs and recurrence

**Fig 6:**
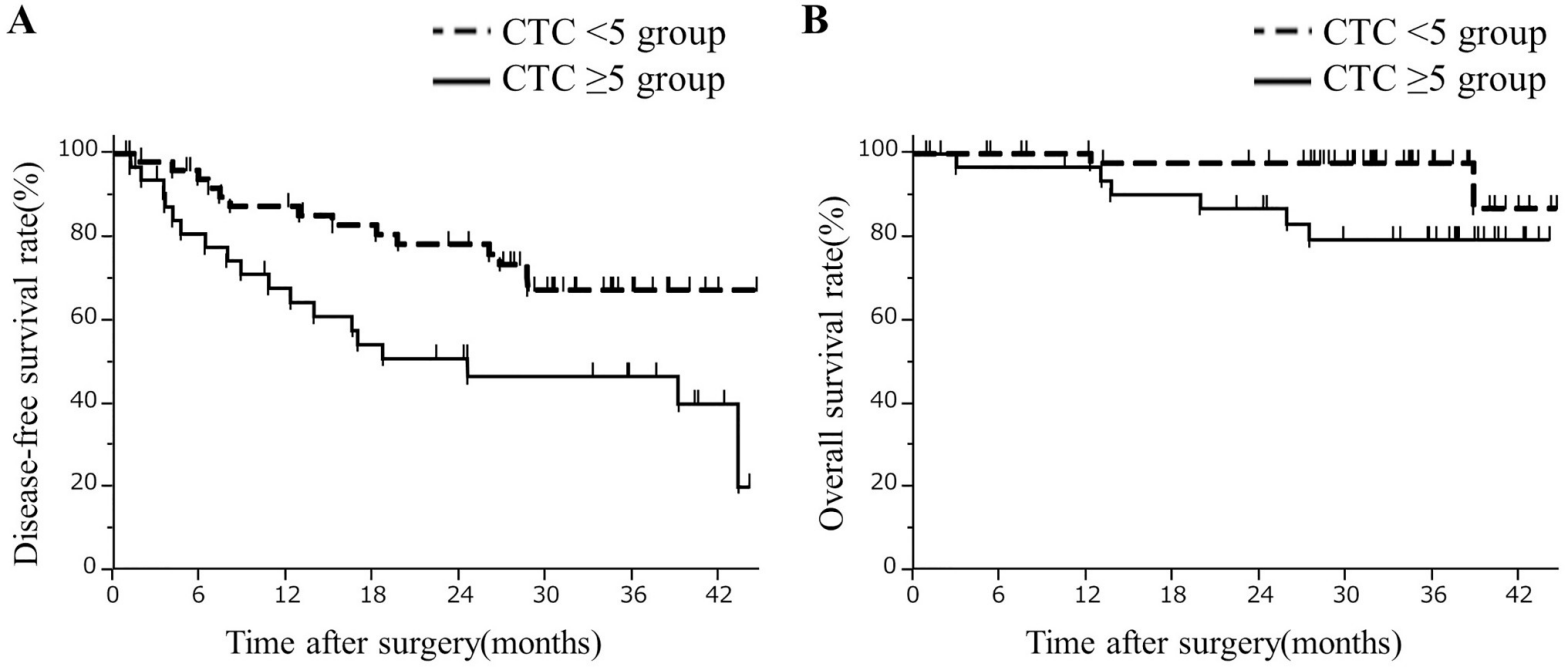
**(A) Comparison of disease-free survival rates after hepatectomy of the CTC ≥5 group to that of the CTC <5 group**. The disease-free survival rates were significantly lower in the CTC ≥5 group than the CTC <5 group (log-rank test; p = 0.015). **(B) Comparison of overall survival rates after hepatectomy of the CTC ≥5 group to that of the CTC <5 group**. The overall survival rates were significantly lower in the CTC ≥5 group than the CTC <5 group (log-rank test; p = 0.047).

The follow-up periods for patients in our study ranged from 1–45 months, with a median of 31.9 months. The 1- and 2-year post-hepatectomy disease-free survival rates were 68% and 51% among patients in the CTC ≥5 group, and 87% and 78% among patients in the CTC <5 group, respectively (p = 0.015, Fig 6A). Disease-free survival rates decreased with an increase in the number of CTCs (S1 Fig). In the CTC ≥5 group, four patients had both liver and distant recurrences and fourteen had liver recurrence only. In the CTC <5 group, one patient had distant recurrence only and thirteen had liver recurrence only. There were no significant differences in the distributions of the recurrence sites (p = 0.100). The 1- and 2-year post-hepatectomy overall survival rates were 97% and 87% among patients in the CTC ≥5 group, and 100% and 98% among patients in the CTC <5 group, respectively (p = 0.047, Fig 6B).

## Discussion

In the present study, CTCs were enumerated via flow cytometry after density gradient centrifugation and immuno-magnetic positive enrichment based upon expression of GPC3 in HCC patients. The present study found that patients in the GPC3-positive CTC ≥5 group had a higher incidence of mPVI, and a lower disease-free survival, and a lower overall survival than those in the GPC3-positive CTC <5 group. Multivariate analysis identified GPC3-positive CTCs ≥5 as an independent predictor of mPVI. To the best of our knowledge, this is the first study on positive enrichment based upon the expression of GPC3, and also the first study of the predictor for mPVI using GPC3-positive CTCs.

Although several studies have demonstrated that the presence of CTCs was significantly associated with prognosis and other clinicopathologic features of HCC [9–14, 16, 17], CTC detection remains in the experimental stages and is not routinely performed in the clinical setting. EpCAM, which is commonly used for the detection of CTCs in other cancers, is only expressed in approximately 35% of HCC cases [7, 8]. In addition, EpCAM-positive circulating epithelial cells have also been detected in patients with benign diseases, resulting in possible false positives [24]. These results highlight the importance of identifying more specific HCC CTC markers. Xu et al. have reported that the asialoglycoprotein receptor (ASGPR) is useful for the detection of HCC CTCs [17]. However, the expression of ASGPR decreases commensurate with the degree of HCC de-differentiation. As poorly differentiated cancer cells are more likely to undergo metastasis than moderately and well-differentiated cancer cells, it follows that ASGPR may be unsuitable for the detection of de-differentiated, metastatic CTCs.

The use of GPC3 as a marker of HCC CTCs can circumvent the aforementioned challenges. Indeed, GPC3 is currently used as a diagnostic biomarker to distinguish HCC from normal hepatic tissue and benign liver tumors [18, 19]. Herein, we provide a novel CTC detection protocol that leverages GPC3-based immuno-magnetic separation and flow cytometry. Flow cytometry is one of the most commonly used for identification of CTCs because of a rapid, sensitive and easy technique. In fact, our CTC detection method can be completed in a short time (within 3 h from density gradient centrifugation to the generation of report). However, it should be noted that the events detected by flow cytometry might contain non-specific events as well as CTCs. Thus, it is considered that one or two non-specific events were detected even in non-HCC patients, and 100% specificity could not be achieved. Importantly, CTCs ≥3 were barely detected in blood samples from non-HCC patients, indicating that our method was not affected by the liver disease status of a patient and could reduce nonspecific events. Moreover, CTCs ≥ 3 could be considered a promising tumor marker for HCC, with a sensitivity of 60.0%. Therefore, this method possesses great potential for enabling the routine clinical analysis of CTCs, as it is sensitive and easy, relatively less expensive, and more rapid compared to the currently used techniques, such as CellSearch system [9–12]. Once clinically validated, it may be applied in any clinical center equipped with MACS and flow cytometry facility.

Patients in the CTC ≥5 group were more likely to present with encapsulated HCC than patients in the CTC <5 group, potentially due to the influence of tumor internal pressure and tumor drainage vessels. Tanaka et al. suggested that the gradient between tumor and portal vein pressure is a causal factor in the dispersion of tumor cells into the portal vein, and that the pressure gradient of encapsulated HCCs is significantly greater than that of non-encapsulated HCCs [25]. In addition, Toyosaka et al. suggested that the vascular channels in the tumor capsule are continuous with the portal vein in encapsulated HCCs [26]. Hence, CTCs from these tumors may be more likely to shed into the blood than those from non-encapsulated HCCs.

It is difficult to detect mPVI before the treatment of HCC, even if recent superior imaging procedures are used during evaluation. It is reported that the risk factors for mPVI are larger tumors, a high AFP, a high DCP, and a macroscopic multinodular type of lesion [27–30]. However, the sensitivity and specificity for predicting mPVI were not satisfactory. We found in our current study that GPC3-positive CTCs ≥5 and high AFP (≥60 ng/mL) were significantly predictive of mPVI. In particular, CTC can predict mPVI with higher sensitivity and specificity. Liu et al. suggested that GPC3 expression in HCC was significantly associated with the presence of vascular invasion [31]. Hence, the presence of GPC3-positive CTC may indicate a high risk of mPVI. Shimada et al. suggested that anatomical liver resection should be considered for patients with mPVI [5]. Therefore, enumeration of CTCs may be useful in determining the range of dissection for the liver. In addition, many studies have reported that the presence of CTCs is a negative prognostic factor for HCC [10–14, 16]. Similarly, the present study showed that patients in the CTC ≥5 group had poorer post-hepatectomy disease-free survival rates and overall survival rates than patients in the CTC <5 group. Thus, GPC3-positive CTCs may be a useful biomarker for HCC patient outcomes. In this study, we did not analyze about liver transplantation or adjuvant therapy. However, Hemming et al. suggested that a preoperative test that could identify vascular invasion or, possibly more importantly, identify CTCs could allow more appropriate selection of patients who would benefit from transplantation [2]. For adjuvant therapy, von Felden et al. suggested that determination of CTC number could be very valuable for selecting patients with the highest HCC recurrence risk for adjuvant regimens [12]. Although the present study could be useful in these areas, including selection of the operative method, liver transplantation, and adjuvant therapy, further study is needed.

In conclusion, we found that glypican-3-positive CTCs are both a predictor of mPVI and correlates with patient prognosis. Therefore, glypican-3-positive CTCs are a useful biomarker for HCC patient outcomes.

## Supporting information

S1 Fig The disease-free survival rates according to the number of CTCs. (TIF)

S1 Table Individual participant data. (XLSX)
