# Supplementary figures and images for: Clinical significance of glypican-3-positive circulating tumor cells of hepatocellular carcinoma patients: A prospective study
Source: PLoS One. 2019 May 29;14(5):e0217586. doi: 10.1371/journal.pone.0217586 (PMC6541303; doi:10.1371/journal.pone.0217586)

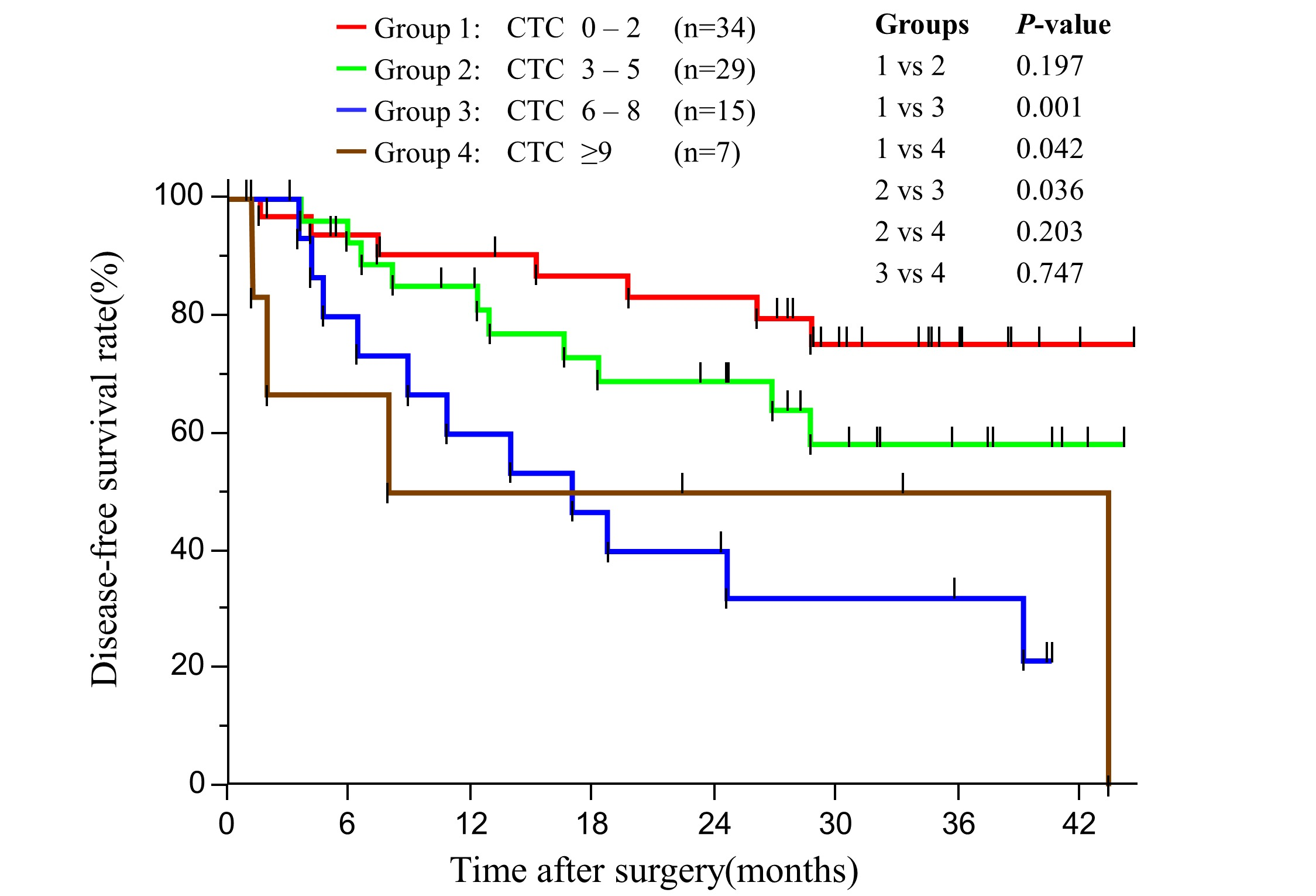

Supplement: S1 Fig — (TIF) [file pone.0217586.s001.tif]
